# PowerAI-Diabetes: Review of glycemic and lipid variability to predict cardiovascular events in Chinese diabetic population

**DOI:** 10.1038/s44324-024-00012-7

**Published:** 2024-07-01

**Authors:** Sharen Lee, Tong Liu, Cheuk To Chung, Johannes Reinhold, Vassilios S. Vassiliou, Gary Tse

**Affiliations:** 1 Diabetes Research Unit, Cardiovascular Analytics Group, PowerHealth Research Institute, Hong Kong, China; 2 Tianjin Key Laboratory of Ionic-Molecular Function of Cardiovascular Disease, Department of Cardiology, Tianjin Institute of Cardiology, Second Hospital of Tianjin Medical University, Tianjin, China; 3 Norwich Medical School, University of East Anglia and Norfolk and Norwich University Hospital, Norwich, NR4 7TJ UK; 4 School of Nursing and Health Studies, Hong Kong Metropolitan University, Hong Kong, China

**Keywords:** Diseases, Endocrine system and metabolic diseases

## Abstract

The aim of this study is to review the predictive value of visit-to-visit variability in glycaemic or lipid tests for forecasting major adverse cardiovascular events (MACE) in diabetes mellitus. Data from existing studies suggests that such variability is an independent predictor of adverse outcomes in this patient cohort. This understanding is then applied to the development of PowerAI-Diabetes, a Chinese-specific artificial intelligence-enhanced predictive model for predicting the risks of major adverse cardiovascular events and diabetic complications. The model integrates an amalgam of variables including demographics, laboratory and medication information to assess the risk of MACE. Future efforts should focus on the incorporation of treatment effects and non-traditional cardiovascular risk factors, such as social determinants of health variables, to improve the performance of predictive models.

## Introduction

Major adverse cardiovascular events (MACE), including acute myocardial infarction, thromboembolic stroke, heart failure and peripheral vascular disease, are major contributors to morbidity and mortality amongst patients with type 2 diabetes mellitus (T2DM). The heightened risk of cardiovascular disease has been well-established for over five decades. A greater incidence of cardiovascular diseases amongst patients with diabetes mellitus, in comparison to their non-diabetic counterparts across all age groups, was first demonstrated by the Framingham Heart Study ≥^1^. The study sparked academic interest early on in exploring the reduction of cardiovascular risk among patients with T2DM. One of the topics explored is the effects and extent of glycemic or lipid control to lower the risks of MACE. In the past, the relationship between glycemic or lipid control and cardiovascular risks was considered to be linear. However, recent evidence has shown that intensive glycemic and lipid control may not be the best way for lowering the risks for MACE in a safe manner^2^. As a result, there has been a shift towards a more patient-centered, individualized approach in the long-term treatment of T2DM^3^. Indeed, new, personalized disease-monitoring parameters were explored over the past decade^4–6^. Besides the glycemic and lipid levels, the temporal fluctuations of glycemic and lipid control were identified as independent risk factors of increased cardiovascular disease risk amongst T2DM patients^7,8^. In the present review, the history and recent advances in medications reducing MACE risk are reviewed. We then discuss the development of PowerAI-Diabetes, an artificial intelligence-enhanced predictive risk model that is specific for the Chinese diabetic population for forecasting MACE. We propose approaches to further improve the performance of predictive models by incorporating treatment effects and non-traditional cardiovascular risk factors, such as social determinants of health variables.

## Glycemic control and the cardiovascular effects of SGLT2i and GLP1A

Hyperglycemia is the foundation to the diagnosis of T2DM, and an integral part of the pathogenesis of MACE amongst patients with T2DM. The diagnosis of T2DM is made by fulfilling any of the following criteria: 1) asymptomatic: fasting blood glucose ≥7.0 mmol/L; 2) 2-h blood glucose after 75 g oral glucose tolerance test ≥11.1 mmol/L; 3) HbA1c ≥ 6.5%; 4) symptomatic hyperglycemia/ hyperglycemic crisis with random blood glucose ≥11.1 mmol/L^9^. High HbA1c was reported to be a significant predictor for fatal and non-fatal cardiovascular diseases in a study of over 18,000 patients in the Swedish National Diabetes Register^10^. Laboratory studies have shown that hyperglycemia induces endothelial dysfunction^11^ and promotes atherogenesis^12^, resulting in a greater atherosclerotic plaque burden and higher vulnerability to rupture^13^. In addition, hyperglycemia is also associated with increases in oxidative stress^14^, which promotes a pro-inflammatory and pro-thrombotic state^15^, with lasting systemic impact after the normalization of glucose concentrations, hence resulting in the significantly increased MACE risk amongst patients with hyperglycemia^16^.

**Fig. 1:**
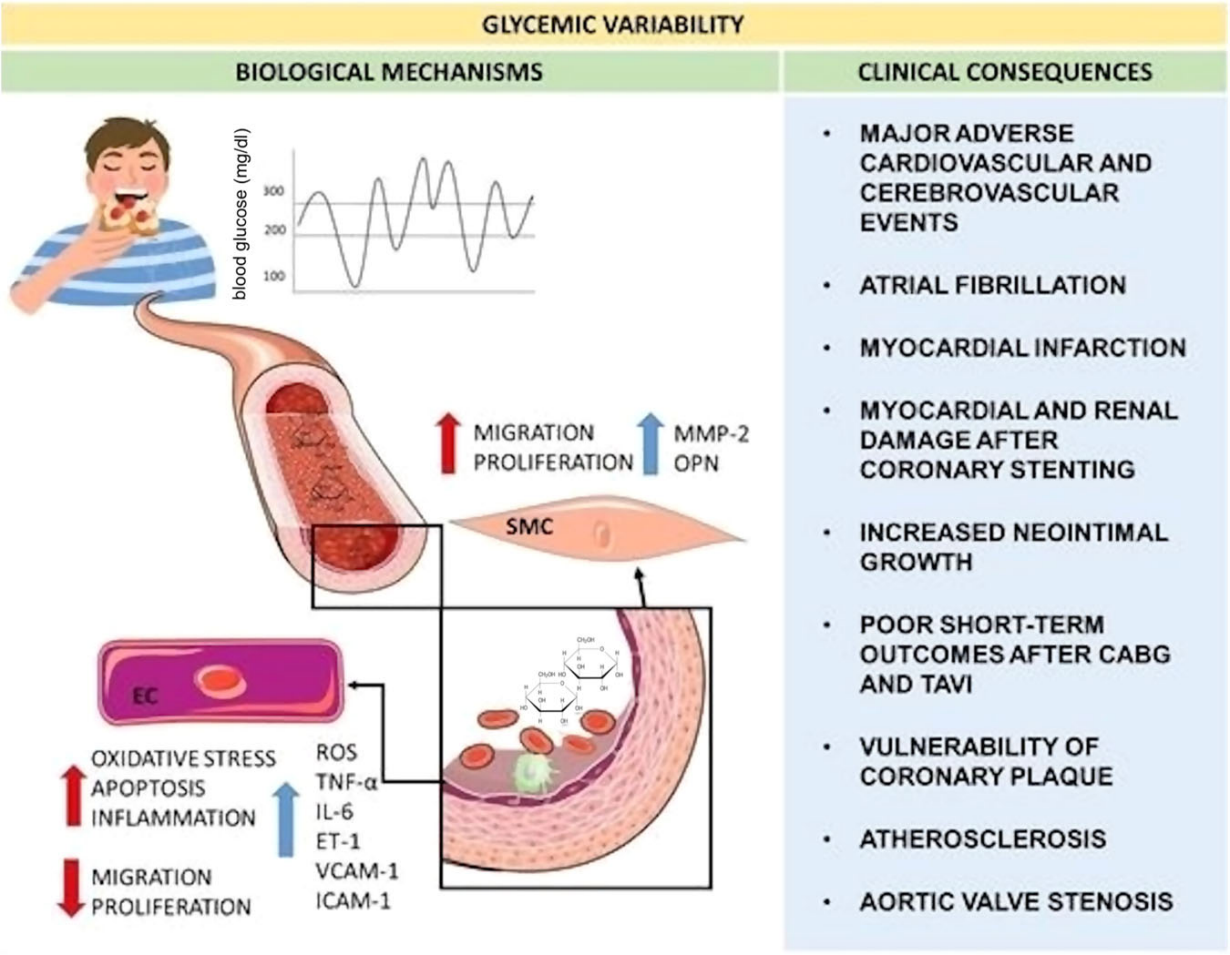
The clinical consequences and biological mechanisms of glycemic variability in relation to MACE events. Reproduced from ref. ^18^ with permission.

In the past, it was thought that the plasma glucose level and MACE are correlated in a linear manner, but previous work has reported U- or J-shaped relationships between measures of glycemic control and MACE or its components^17^. A further elaboration on the relationship between glycemic variability with MACE is illustrated in Fig. 1^18^. The United Kingdom Prospective Diabetes Study (UKPDS) was one of the first landmark studies that showed patients on more intensive glycemic control to have a significantly lower risk for microvascular (but not macrovascular) adverse events^19^. However, subsequent studies raised questions about the attainability of an intensive glycemic target. In the Action to Control Cardiovascular Risk in Diabetes (ACCORDS) trial, the mortality rate was significantly higher in the group with intensive glycemic control (hazard ratio 1.22, *p* = 0.04) than their counterparts in the standard glycemic control group, with a greater incidence of significant weight gain and hypoglycemia, resulting in early termination of the trial^2^. It was found that persistent hypoglycemia, albeit asymptomatic, may be pro-arrhythmic for patients with T2DM and high cardiovascular risks^20^. In addition, a significant association between severe hypoglycemia and all-cause mortality was established in the double-blind Trial Comparing Cardiovascular Safety of Insulin Degludec vs Insulin Glargine in Patients with Type 2 Diabetes at High Risk of Cardiovascular Events (DEVOTE) study^21^. As a result, an individualized approach has been adopted by recent guidelines. The 2022 consensus document from the American Diabetes Association (ADA) and the European Association for the Study of Diabetes (EASD) states that whilst HbA1c ≤ 7% is a reasonable glycemic target for most non-pregnant adults, a lower HbA1c target could be sought for if it can be attained safely without adverse health effects, and a higher target is appropriate for patients with limited life expectancy, poor premorbid state or advanced complications^3^.

The introduction of novel classes of anti-diabetic agents facilitates the attainment of glycemic control safely with cardiovascular protective effects. Sodium-glucose cotransporter-2 inhibitors (SGLT-2i) are the newest antidiabetic agent that lowers blood glucose by promoting urinary glucose excretion in the proximal tubular cells in the kidneys. Significant cardio-renal protective effects were demonstrated across trials^22,23^, with the greatest benefit in the reduction of hospitalization for heart failure and renal outcomes^24^. The combination of preload and afterload reduction, attenuation of cardiac fibrosis, and improvements in myocardial metabolism protects the cardiovascular system against adverse events directly^25,26^. Most notably, sodium-glucose cotransporter-2 inhibitors (SGLT2is) are indicated for diabetic and non-diabetic patients with heart failure, across the spectrum of left ventricular ejection fractions^27,28^.

Glucagon-like peptide-1 receptor agonists (GLP1a) are another new class of antidiabetic agents with potent glucose-lowering and weight-reducing effects, and acceptable safety profiles^29^. In addition to augmenting the secretion of insulin in the pancreatic islet cells, GLP1a also slows gastric emptying, thus improving the attainment of glycemic targets^30,31^. GLP1A use is linked to lower risks of MACE of T2DM patients with established cardiovascular diseases, or high cardiovascular risk^32^. Liraglutide has been reported to be more effective in achieving the target HbA1c in comparison to a sulphonylurea or dipeptidyl peptidase-4 inhibitor (DPP4i) with a lower risk in the composite outcome of MACE, revascularization, and heart failure/ unstable angina requiring hospitalization by The Glycemia Reduction Approaches in Diabetes: A Comparative Effectiveness Study (GRADE) multicenter open-label randomized controlled trial^33^.

## Lipid control and the cardiovascular effects of statin and non-statin drugs

Low-density lipoprotein cholesterol (LDL-C) drives atherogenesis via a multitude of mechanisms including the induction of endothelial inflammatory response and promotion of plaque rupture. Besides reducing the formation of atherosclerotic plaques, studies have shown that the lowering of LDL-C stabilizes existing plaques by changing the plaque content^34^. By increasing the thickness of the fibrous cap, the atheromas have a lower risk of rupture and subsequent thrombosis, therefore ultimately reducing the risk of MACE^35^. Similarly, elevated triglyceride also marks for an increased risk for MACE in patients with T2DM since it represents both the concentration of atherogenic remnant cholesterols in the circulation and the tissue resistance against insulin^36^. It has also been reported that the use of lipid-lowering therapy can lead to the regression of carotid artery stenosis, suggesting that intensive lipid control may be able to halt or even reverse the progression of atherosclerotic cardiovascular disease^35^. As a result, the attainment of lipid control has become an integral part of the treatment goal for T2DM.

Advancements in strategies of lipid control played an important role in the improvement of cardiovascular risk reduction amongst T2DM patients. In a meta-analysis that included 14 randomized controlled trials on the use of lipid-lowering agents amongst patients with T2DM, it was shown that a 1 mmol/L reduction in LDL-C reduces the risk of major vascular events by 21%^37^. As reported by the Treatment to New Targets (TNT) study, lowering LDL-C to <1.99 mmol/L, below the recommended LDL-C threshold of 2.59 mmol/L at the time, resulted in a 25% reduction in the risk of MACE^38^.

In the past, statins were the major lipid-lowering agents with benefits derived from both their lipid-lowering and anti-inflammatory effects. The primary targeted cell types include vascular endothelial cells, chemokines and immune cells to modify cholesterol homeostasis^39^. With the development of non-statin lipid-lowering strategies, more aggressive LDL-C targets became achievable. In the Improved Reduction of Outcomes: Vytorin Efficacy International Trial (IMPROVE-IT) study, ezetimibe was able to further lower LDL-C by 24% in patients with relatively lower baseline LDL-C levels, with greater cardiovascular protective effects noted amongst users with T2DM^40^. Evidence for the cardiovascular protective effects of PCSK9i is also highly promising^41^.

By contrast, evidence on the cardiovascular-protective effects of triglyceride-lowering therapies is more limited. Fibrates, which lowers triglyceride level by peroxisome proliferator-activated receptor (PPAR) modulation, expressed predominately in the liver, kidney and muscle. The recent Pemafibrate to Reduce Cardiovascular Outcomes by Reducing Triglycerides in Patients with Diabetes (PROMINENT) multinational, double-blinded, randomized controlled trial showed that pemafibrate does not lower the incidence of MACE despite its efficacy in the reduction of cholesterol levels^42^. Therefore, contrary to LDL-C management, the control of hypertriglyceridemia is not a prioritized treatment target.

## Factors contributing to temporal variability and MACE risk

**Fig. 2:**
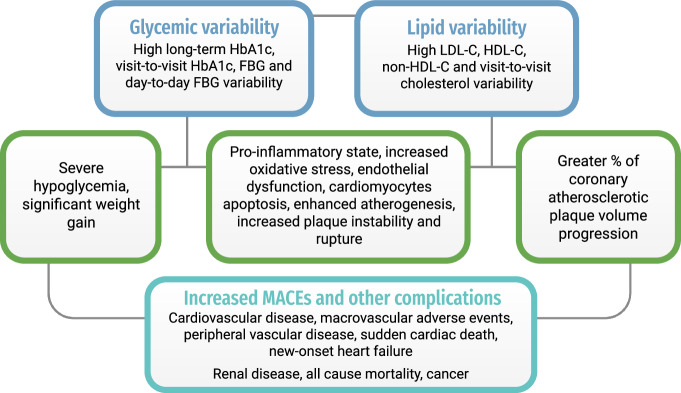
Summary of pathogenic mechanisms underlying glycemic/ lipid variability and major adverse cardiovascular events.

With a shift towards a personalized approach in the management of T2DM, there has been a call for novel, individualized disease-monitoring parameters in the evaluation of cardiovascular risks among patients. Some parameters that are commonly identified include blood pressure, male gender and a family history of cardiovascular diseases^43^. The shift in personalized medicine may be attributed to the rapid development of technological breakthroughs such as genetic testing and Continuous Glucose Monitoring systems that enables refined glycemic control and empower patient engagement^44^. Some existing approaches for personalized T2DM management may include custom dietary plans based on culture and glycemic index, tailored lifestyle interventions, pharmacogenomics-informed therapy to optimize drug effectiveness^45–47^. The predictive value of time-varying parameters in glycemic and lipid control has also become a growing topic of interest over the past decade. The explanations for the relationships between increased glycemic or lipid variability and higher risks of MACE are illustrated in Fig. 2.

Independent of HbA1c and glucose levels, increased glycemic variability has been reported to increase the risk of microvascular, macrovascular complications and MACE^48^. A meta-analysis of 13 studies demonstrated that long-term glycemic variability, represented by HbA1c variability, is associated with a higher risk of cardiovascular disease, macrovascular events, renal disease and all-cause mortality^49^. In a *post-hoc* analysis of the visit-to-visit HbA1c variability and fasting glucose of the Action in Diabetes and Vascular Disease (ADVANCE) trial, raised fasting glucose and HbA1c variability are both associated with an increased risk for macrovascular adverse events^50^. Similarly, a *post-hoc* analysis of the Veteran Affairs Diabetes Trial demonstrated a significant positive association between fasting glucose variability and cardiovascular disease after adjusting for risk factors, including mean fasting glucose^51^. To address the lack of adjustments for confounders in existing evidence, machine learning techniques were introduced to account for the latent interactions between glycemic variability and other cardiovascular risk factors. A recent study on more than 25,000 T2DM patients using insulin demonstrated that higher HbA1c and lipid variability was associated with elevated risk of peripheral vascular disease and mortality. The study also applied regularized and weighted random survival forest models to account for interacting relationships improves the accuracy of prediction to a c-statistic of over 87%^52^. Moreover, HbA1c and lipid variability were predictive of sudden cardiac death in patients with advanced stages of T2DM requiring insulin therapy^53^. In an expanded cohort study of T2DM patients, a multivariable model incorporating indices of inflammation, HDL-cholesterol, total cholesterol, triglyceride, HbA1c and fasting blood glucose (FBG), measures of variability of both HbA1c and FBG showed a c-statistic of 73% when using a score-based predictive risk model, which was lower compared to a value of 86% and 87% when using random survival forests and deep survival learning models, respectively^54^.

The relationship between high HbA1c variability and MACE may extend to other diseases such as cancer^55^. The pro-inflammatory state and increased oxidative stress environment produced by fluctuating levels of glycemia are associated with increased risks of MACE, which may be partly mediated through endothelial dysfunction^56,57^. Furthermore, preclinical evidence shows that high glycemic fluctuation also induces tissue oxidative stress and can lead to increased apoptosis of cardiomyocytes^58^.

Besides long-term glycemic variability, the predictive value of short-term day-to-day variability has also been explored. The Degludec vs Insulin Glargine in Patients with Type 2 Diabetes at High Risk of Cardiovascular Events (DEVOTE 2) trial showed that the standard deviation of the self-monitored blood glucose over three days was associated with MACE and all-cause mortality^48^. Both day-to-day FBG variability and HbA1c variability were associated with severe hypoglycemia^59^, possibly suggesting hypoglycemia as a mediator for MACE. The calculation of the standard deviation allowed for the separation of day-to-day variability from overall mean glycemic control, as the latter may be influenced by the treat-to-target regimen.

Whilst T2DM patients were known to have an increased variability of plasma lipid level, it was only recently that its predictive values for cardiovascular risks were examined. Studies have shown that high LDL-C, high-density lipoprotein cholesterol (HDL-C) and non-HDL-C variability were independent risk factors for cardiovascular disease in T2DM after adjusting for confounding variables^56,60^. In a study of over 125 000 patients with T2DM under primary care in Hong Kong, it was noted that lipid variability was a significant predictor for cardiovascular disease, with the strongest predictors being LDL-C variability, particularly amongst the younger age group between 45–54 years old^61^. Moreover, recent work has reported significant relationships between visit-to-visit cholesterol variability and long-term risks of new-onset heart failure and major adverse cardiovascular outcomes also in patients under primary care in Hong Kong^62^ as well as South Korea^63,64^. Whilst the pathogenic mechanism remains unclear, it was hypothesized that increased LDL-C variability increases plaque instability due to the promotion of macrophage activation to form cholesterol core in atheromas, ultimately leading to plaque rupture^65^. The atherogenic physiology under high lipid variability was further supported by the recent report of a positive association between the variability of LDL-C/ total cholesterol to HDL-C ratio and the percentage of coronary atherosclerotic plaque volume progression^66^.

## PowerAI-diabetes: Chinese-specific AI predictive model

**Fig. 3:**
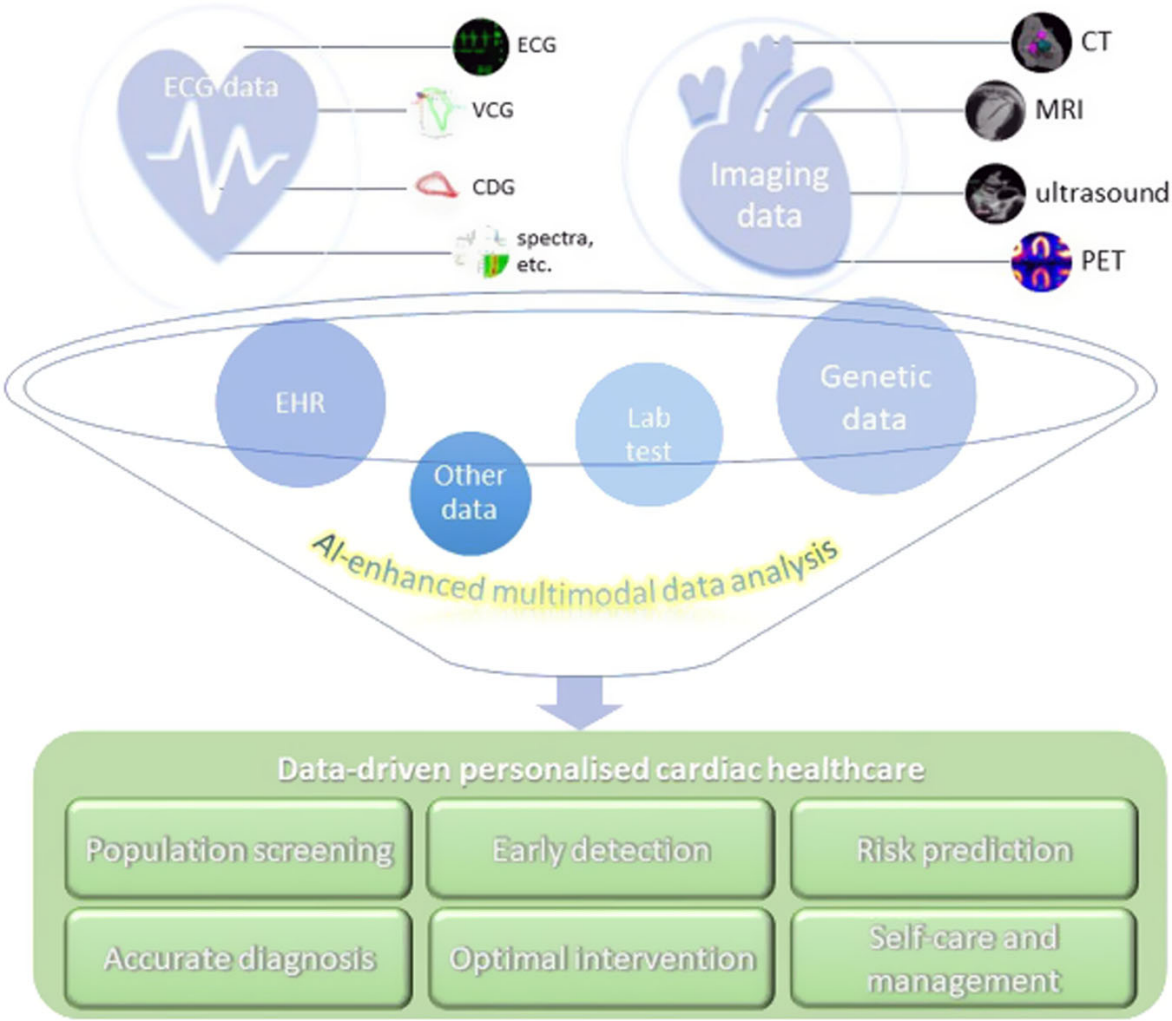
Approach for model development. Our model can accurately predict nine different diabetes-related complications (neurological, ophthalmological, CKD, dementia, osteoporosis, peripheral vascular disease, ischemic heart disease, atrial fibrillation and heart failure). Reproduced from ref. ^67^ with permission.

**Fig. 4:**
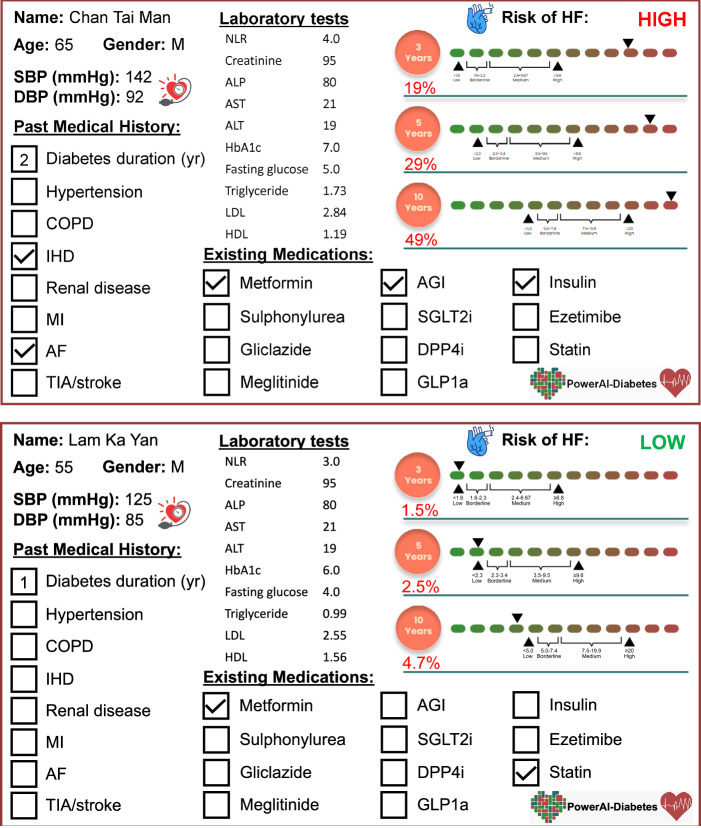
Dashboard showing estimation of 3-, 5- and 10-year risk of heart failure in a high-risk patient (*top*) and a low-risk patient (*bottom*).

We developed a Chinese-specific AI-driven predictive model for diabetic patients^67^, which we termed PowerAI-Diabetes. Our model is the first globally to incorporate both lipid and glycemic variability for risk prediction and has the capability to predict 9 complications. The multimodal model development of the PowerAI-Diabetes algorithm is illustrated in Fig. 3. A high-risk and a low-risk patient for developing heart failure at 3-, 5- and 10-year timepoints are shown in the top and bottom panels of Fig. 4, respectively.

**Fig. 5:**
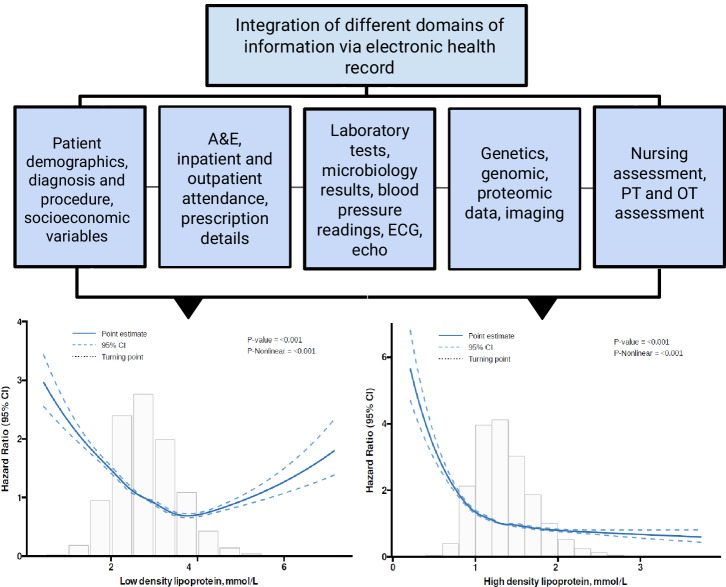
Integration of massive amounts of data from different domains to develop state-of-the-art models enhanced by artificial intelligence.

The application of AI has revolutionized predictive analytics, from disease diagnosis^68^ to CVD risk prediction^69^. In Hong Kong, our team’s recent systematic review has identified a striking rise in the number of big data studies since 2015, reaching a total of 120 papers published in 2022 alone^70^. In PowerAI-Diabetes, we included baseline demographics, disease status, laboratory tests, and medications at baseline. Integration of massive amounts of data from different domains (Fig. 5), ranging from non-invasive tests such as electrocardiography and echocardiography, to invasive tests such as procedure findings, can further enhance risk prediction^71^. Our team found that data mining of ECG features can predict a variety of outcomes such as atrial fibrillation and heart failure^72^. Other data fields such as social determinants of health metrics^73^, nutritional indices, and functional assessment by different multidisciplinary team members all provide important information to improve risk prediction. A truly comprehensive model would thus consider not only narrow medical determinants, but broader determinants^74,75^, incorporating polygenic^76^, polysocial^77^ as well as polyexposure^78^ risks.

The model was constructed using information from a cohort of 25,186 patients, applying random survival forest to process and learn the dataset. The tree ensembling approach was applied to estimate the forest hazard survival function and to allocate equal weights on multiple survival trees. To take into consideration the potential heterogeneity in the data, the best results were selected after testing multiple values for the weighting and regularization parameters. Subsequently, this approach allows for the prediction of MACE outcomes and other diabetic complications. In addition, the variable importance approach was also adopted to estimate the relative predictive value of each risk factors^52^.

He et al. developed a machine learning algorithm that predicts recurrent risk of MACE occurrence amongst the Asian population, demonstrating superior performance to existing benchmark risk score models. While this model did not include the measurement of HbA1c due to data limitation, our model has taken this variable into consideration^79^. Hsu et al. constructed a multivariate Cox regression model using data from a Taiwan cohort with T2DM to estimate the association between glycemic variability and MACE. The study’s results of the strong relationship between higher glycemic variability and increased risk of MACE occurrence aligns with our findings. However, our PowerAI-Diabetes model extrapolates further from the results of this study to be able to potentially perform risk stratification for patients using AI-derived approaches^80^.

The current model has some limitations, most notably for not including important risk predictors. For example, even though hypertension, a main driver of CVD risk^81^, was included, real-time blood pressure values were not, leaving residual risk unaccounted for. Moreover, smoking status was missing from the current dataset. This notwithstanding, the model was unable to stratify the outcomes based on gender, age and presenting symptoms due to limited data. Moreover, given the cohort of patients was recruited in 2009, the anti-diabetic medications SGLT2i, DPP4i and GLP1A were not yet available. We are now updating the model to consider blood pressure, blood pressure variability, smoking status, as well as treatment effects of the anti-diabetic medication classes SGLT2i, DPP4i and GLP1A, as well as lipid-lowering drugs such as PCSK9i. In the future, we hope to develop the model to be able to further stratify data based on gender and age, as well as stratify the predictability of variables based on asymptomatic patients or patients with sub-clinical symptoms. To corroborate, further external validation in comparison with other predictive models is warranted to better determine the precision and accuracy of the PowerAI-Diabetes approach.

## Big data analytics for model development and implementation

With a drive towards big data analytics to achieve personalized care, territory-wide electronic health records data provide a vast amount of multi-modality information that can facilitate early diagnosis and risk stratification. Whilst various CVD risk assessment tools are widely available, the majority were developed using Western population data. When applied to other demographics such as Chinese populations, the predictive performance of these models is lower, with low-risk patients classified as high-risk, and vice versa. This is the long-standing reference class problem which is faced across various clinical settings in healthcare^82^. For a more detailed discussion, the reader is kindly referred to our Editorial published in 2018^83^. The use of big data analytics of routinely collected electronic health records provide major opportunities for developing highly accurate models, given the availability of large amounts of multi-modality data involving vast amounts of data fields from large sample sizes. Coupled with the ease of storage and retrieval of decades of longitudinal data, accurate models predicting disease onset, trajectory and numerous adverse outcomes can be developed. Such models would be highly generalizable and universally applicable to the population from which they were developed. Using real-world clinical practice data rather than randomized controlled trial data would extend the applicability of the risk models from limited age ranges with few comorbidities to patients with a full range of ages and comorbidity burden.

Furthermore, with the capture of multiple measurements for each data field, it is possible to consider not only time-invariant but also time-varying parameters, allowing more accurate risk predictions. For example, we reported that for COPD patients, treatment of cumulative systemic steroid dose as a time-varying measure can better predict the risks of CVDs and other adverse outcomes^84,85^. A natural extension of using longitudinal data would be dynamic risk prediction, whereby not only a single value at baseline but newer values, or multiple values obtained beyond the baseline period, are used as input to provide updated risk estimates. We have found that visit-to-visit variability across multiple years before baseline could further improve risk prediction beyond using a single baseline value or mean value of multiple measurements^86^. It is thus conceivable that recalculation of these measures of variability can provide updated risks.

For future developments, the current big data models can be improved by further individualizing to different diseases. We have developed the first-in-world, AI-enhanced, Chinese-specific CVD predictive risk model for the general population as described previously^87^, and the diabetes-specific CVD risk model described here. The next step would be developing CVD risk models for other important conditions, such as cancer and COPD. The reason is that for each disease, a different set of input variables can better predict CVD risk. For example, in COPD, our team found that the number of exacerbations at baseline and respiratory medications can influence CVD risk. Similarly, for cancer, the disease itself as well as different anti-cancer drugs exert cardiotoxic effects and thus alter CVD risk^88^.

There are important considerations for the future. Effective models must be implemented and used routinely in order to make a difference to patient outcomes. Evidence-based decision-making algorithms that are easy to follow are needed to guide clinical management for clinicians. Different stakeholders must be engaged, including clinicians, healthcare administrators, systems analysts, end-users and patients can provide valuable input during the cycle of model development, evaluation, refinement and implementation.

## Conclusion

To conclude, glycemic and lipid level targets are an integral part of disease management in T2DM, particularly in terms of the risk management of MACE. With the call for an individualized approach in the management of T2DM, novel disease-monitoring parameters were explored. The temporal variability of glycemic and lipid parameters was noted to be of significant predictive value in the risk stratification of cardiovascular diseases, particularly amongst patients with T2DM. Whilst more evidence was needed to elucidate the pathogenic mechanism underlying high glycemic and lipid variability on increased cardiovascular risk, temporal variability of glucose and lipid levels were incorporated into cardiovascular risk stratification models such as the PowerAI-Diabetes to improve the accuracy of predictions. There may be promising potential to use PowerAI-Diabetes in taking preventive measures to limit the occurrence of MACE in diabetic patients in a real-life clinical setting. We anticipate that the model can improve upon patient care and generate data that is generalizable to diabetic patients of the Chinese population.
